# Beneficial Effect of Dietary Diversity on the Risk of Disability in Activities of Daily Living in Adults: A Prospective Cohort Study

**DOI:** 10.3390/nu12113263

**Published:** 2020-10-25

**Authors:** Jian Zhang, Ai Zhao, Wei Wu, Zhongxia Ren, Chenlu Yang, Peiyu Wang, Yumei Zhang

**Affiliations:** 1 Department of Nutrition and Food Hygiene, School of Public Health, Peking University, Beijing 100191, China; zhangjian92@pku.edu.cn (J.Z.); wennie0616@163.com (W.W.); ren_zhongxia@pku.edu.cn (Z.R.); yangchenluwork@126.com (C.Y.); 2 Vanke School of Public Health, Tsinghua University, Beijing 100091, China; aizhao18@tsinghua.edu.cn; 3 Department of Social Medicine and Health Education, School of Public Health, Peking University, Beijing 100191, China; wpeiyu@bjmu.edu.cn

**Keywords:** dietary diversity, activities of daily living, cohort study, adults

## Abstract

Disability in activities of daily living (ADL) is common in elderly people. Dietary diversity is associated with several age-related diseases. The evidence on dietary diversity score (DDS) and ADL disability is limited. This study was based on the China Health and Nutrition Survey. Prospective data of 5004 participants were analyzed. ADL disability was defined as the inability to perform at least one of the five self-care tasks. Cox proportional regression models were conducted to estimate the association of cumulative average DDS with the risk of ADL disability. Logistic regression models were performed to estimate the odds ratios for the average DDS, the baseline DDS, and the recent DDS prior to the end of the survey in relation to ADL disability, respectively. The results indicate that higher average DDS was associated with a decreased risk of ADL disability (T3 vs. T1: hazard ratio 0.50; 95% confidence interval 0.39–0.66). The association was stronger among participants who did not had comorbidity at baseline than those who did (P-interaction 0.035). The average DDS is the most pronounced in estimating the association of DDS with ADL disability of the three approaches. In summary, higher DDS has beneficial effects on ADL disability, and long-term dietary exposure is more preferable in the investigation of DDS and ADL.

## 1. Introduction

The world’s population is aging at a faster speed than it used to be according to a report from the World Health Organization [1]. It is predicted that the number of older people worldwide would more than double by 2050 [2]. The aging process is accompanied by declines in functional capacity and cognition [3,4]. Disability in older people is associated with low quality of life [5], increased burden on caregivers [6], rise in morbidity and mortality [7], and elevated health care costs [8], which finally put more burdens on the whole society.

Activities of daily living (ADL) is an index to represent the capability of skills essential to manage basic physical needs, comprised of the following areas: grooming/personal hygiene, dressing, toileting/continence, transferring/ambulating, and eating [9]. The prevalence of ADL disability in Chinese individuals aged 60 years and above was 10.03% in 2015 [10]. Actions aimed at preventing ADL dependence may reduce the burden on older people themselves, caregivers, and the health care system [11]. Several factors are reported to be associated with ADL disability, including gender, slow gait speed, weight loss, muscle strength, etc. [5,12]. In addition to that, diet and nutrition are found to have an impact on ADL or functional capacity. Previous prospective studies indicated that higher consumption of dairy products is associated with a lower risk of functional disability [13]. Nutrients, such as vitamin D [14] and dietary fiber [15], are also reported to be related to ADL.

Dietary diversity is an important aspect of our diet. Keeping a diverse diet is one of the key recommendations of Chinese dietary guidelines [16]. Epidemiological studies showed that a higher dietary diversity score (DDS) is inversely associated with some age-related diseases, such as diabetes [17], cognitive decline [18]. Additionally, it has been shown that adherence to a diverse diet reduced the risk of death [19,20]. However, prospective evidence on DDS and ADL disability is limited. In this study, we prospectively analyzed the effect of DDS on the risk of ADL disability in a prospective cohort study.

## 2. Materials and Methods

### 2.1. Study Population

**Figure 1:**
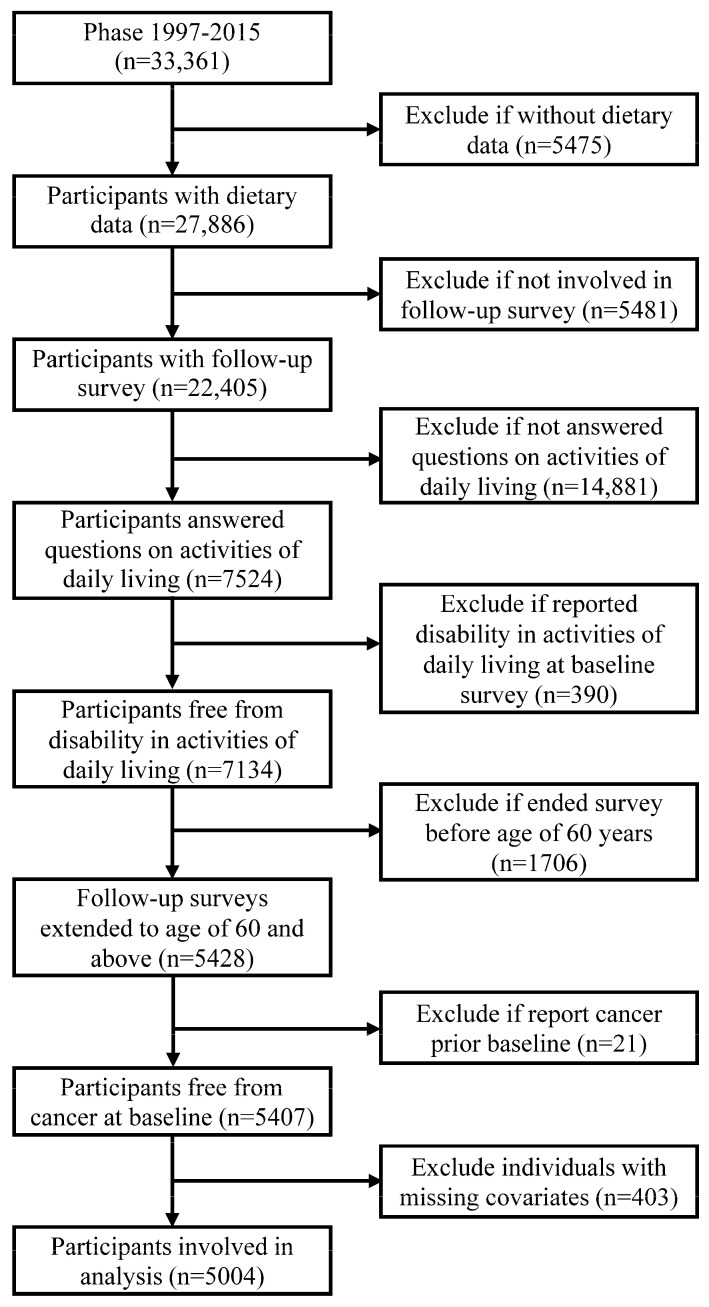
Flow chart of sample selection.

This study was based on the China Health and Nutrition Survey (CHNS). Details about the CHNS has been described elsewhere [21]. In brief, the CHNS is an open cohort study, and participants from twelve geographically diverse areas of China were involved in this study. The CHNS was designed to understand the health and nutrition of the Chinese and how they are affected by social transformation. It was initiated in 1989, and follow-up surveys were conducted in 1991, 1993, 1997, 2000, 2004, 2006, 2009, 2011, and 2015. Data collected in phase 1997 and beyond were used in the present study. The inclusion criteria included answered the ADL survey and aged 60 years and above at the end of the survey (report of ADL disability, loss to follow-up, the phase of 2015, whichever occurred first). The exclusion criteria included absent from dietary survey, report of ADL at baseline survey, report of previously diagnosed cancer at baseline survey, and missing values on covariates. In the end, 5004 participants were included this study (Figure 1). The CHNS was approved by institutional review boards at the University of North Carolina at Chapel Hill and the National Institute of Nutrition and Food Safety, Chinese Center for Disease Control and Prevention. All participants gave written informed consent before they participated in the survey.

### 2.2. Dietary Survey and Dietary Diversity Score

Participants’ diet intakes in the past 3 days were recorded by dietary recall and household food weight inventory. Details about the dietary survey process have been published [22]. All food items that a participant consumed in a 24 h period were divided into eight food categories (cereals and tubers, vegetables, fruits, meat, aquatic products, eggs, soybeans and nuts, and dairy products). Intake of any food from each of the food categories will add one point to DDS, with a total score of eight. Average daily DDS was calculated for each participant at each phase. Cumulative average DDS across phases before the end of the survey (report of ADL disability, loss to follow-up, the phase of 2015, whichever occurred first) was computed to represent long-term diet exposure. Subsequently, average DDS was grouped into tertiles from low to high (T1: 1.33–3.25; T2: 3.27–4.03; T3: 4.06–8.00). Besides, participants’ nutrient intakes were estimated according to the Chinese food composition tables, and cumulative average daily intakes were also computed.

### 2.3. Ascertain of Disability in Activities of Daily Living

In phase 1997, 2000, 2004, 2006, and 2015, participants aged 55 years and above were asked whether she or he could finish some self-care tasks (standing up after sitting for a long time, dressing, toileting, bathing, and feeding). For each question, participants were asked “Do you have any difficulty doing this?”: no difficulty; having some difficulty, but can still do it; need help to do it; cannot do it at all; and unknown. Participants who chose “need help to do it” or “cannot do it at all” in at least one of the five activities were defined as ADL disability [23]. 

### 2.4. Covariates

Information on sociodemographic characteristics, lifestyle behaviors, and personal health and medical history were collected in the CHNS. Baseline age, gender, living region, residency, education level, per capita household income, smoking status, alcohol consumption, physical activity, body mass index (BMI), and medical history (cancer, hypertension, diabetes, stroke, and myocardial infarction) were obtained from the phase at entry. 

Per capita household income was estimated at each phase, and missing values for per capita household income was replaced by the medians of each survey site. Income was grouped into tertiles and was labeled as low, middle, and high at each phase. Physical activity was measured in terms of metabolic equivalent of task (MET)-hours per week [24]. Missing values for height and weight were replaced by the means of two adjacent surveys. BMI was computed as weight (kg)/(height (m))^2^. Since blood pressure was measured, participants who had been previously diagnosed with hypertension or with systolic blood pressure ≥ 140 mmHg and/or diastolic blood pressure ≥ 90 mmHg were all regarded as hypertensive patients. Other medical histories were based on the self-report from participants. Comorbidity was defined as having at least one of the four diseases (hypertension, diabetes, stroke, and myocardial infarction) at the baseline survey. 

### 2.5. Statistics

Continuous variables were presented as means and standard deviations or medians and quartiles according to the distribution of data; categorical variables were presented as percentages. Normally distributed continuous variables were tested between groups by one-way analysis of variance, for categorical variables, chi-square tests were performed for difference across groups. To estimate the trends of nutrient intakes across DDS tertiles (T1, T2, T3), linear regression models were conducted. We assigned the midpoint values of DDS tertiles and treated the variables as continuous in the linear regression model. Values of nutrient intakes were transformed to log to reach normality.

Person years were measured from baseline survey date until the last survey date (report of ADL disability, loss to follow-up, or the phase of 2015, whichever occurred first). To assess the effect of DDS on the risk of ADL disability, hazard ratios (HRs) for DDS and ADL disability were estimated by Cox proportional hazard regression models. Multivariate models were conducted. In the first model, covariates including age at entry (continuous), gender (men or women), living region (southern or northern China), residency (urban or rural), income (low, middle, or high), and education level (primary school and below or middle school and higher) were adjusted. In the second model, smoking status (smoker or not), physical activity (≤100 or >100 MET-hours/week), BMI (continuous), and comorbidity (yes or no) were additionally adjusted. 

Sensitivity analyses were performed by (1) excluding individuals whose follow-up time was less than 5 years and (2) the adjustment of alcohol consumption (regular consumer or not) and phase at entry (1997, 2000, 2004, 2006, 2009, or 2011). Subgroup analyses were conducted in participants with different baseline characteristics (gender, living region, age at entry (≤65 or >65 years old), and comorbidity).

To assess the short-term and the long-term effects of DDS on ADL disability, we analyze the DDS value reported in different surveys with the odds of ADL disability. Individuals involved in three or more dietary surveys were included. Logistic regression models were performed to estimate the odds ratios for the average DDS across phases, the baseline DDS, and the recent DDS prior to the end of the survey in relation to ADL disability, respectively. 

All the statistics were conducted in R 4.0.2. All *p* values were two-sided, and statistical significance was defined as *p* < 0.05.

## 3. Results

### 3.1. Dietary Diversity Score and Baseline Characteristics

The average DDS among participants was 3.78 ± 1.04 (ranged from 1.33 to 8.00). Table 1 presented the baseline characteristics of participants according to DDS tertiles. Compared with individuals in the lowest tertile of DDS, those scoring higher in DDS were older when they entered the survey, had a higher proportion of men, had higher BMI, were more likely to live in southern China and urban areas, had higher education level and income, were less likely to be a smoker, and had lower physical activity. The distribution of DDS was similar in regular alcohol drinkers and the others. 

### 3.2. Dietary Diversity Score and Nutrient Intakes

DDS was positively associated with intakes of protein, fat, dietary fiber, vitamin A, riboflavin, niacin, vitamin C, vitamin E, calcium, phosphorus, potassium, magnesium, iron, zinc, and selenium. Meanwhile, higher DDS was inversely associated with intakes of carbohydrate, sodium, and manganese (Table 2).

### 3.3. Dietary Diversity Score and Disability in Activities of Daily Living

During a median follow-up of 9 years (ranged from 2 to 18; total person years: 52,297), 601 ADL was reported. Absolute ADL rates according to DDS tertiles (from low to high: T1, T2, T3) were 14.4, 10.5, and 8.7 per 1000 person years, respectively. After the adjustment of covariates, higher DDS was associated with decreased risk of ADL disability (Table 3).

### 3.4. Sensitivity Analyses

In the sensitivity analysis, the association of DDS with ADL disability did not change by excluding individuals whose follow-up time was less than 5 years, or additional adjustment of alcohol consumption and phase at entry (Table S1).

### 3.5. Subgroup Analyses

**Figure 2:**
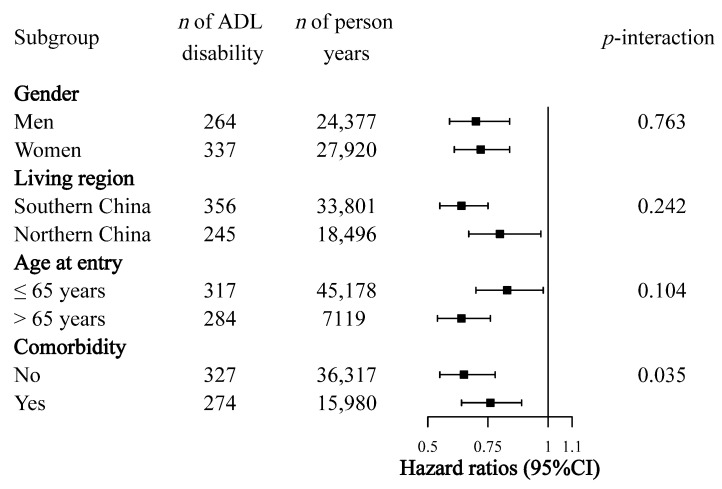
Subgroup analysis of association of continuous dietary diversity score (DDS) with disability in activities of daily living (ADL). Hazard ratios were estimated by Cox proportional regression models. Multivariate models were adjusted for age at entry (continuous), gender (men or women), living region (southern or northern China), residency (urban or rural), income (low, middle, or high), education level (primary school and below or middle school and higher), smoking status (smoker or not), physical activity (≤100 or >100 metabolic equivalent of task-hours/week), body mass index (continuous), and comorbidities (no or yes). Analyses within subgroups were adjusted for all other covariates.

The significant association between DDS and ADL disability was observed in all subgroups based on baseline characteristics, including gender, living region, age at entry, and comorbidity. However, the association was more pronounced in participants living in southern China, participants aged over 65 years at entry, and participants without comorbidities than the others. A significant interaction term between DDS and comorbidity was observed (Figure 2).

### 3.6. Dietary Exposure Measures

**Figure 3:**
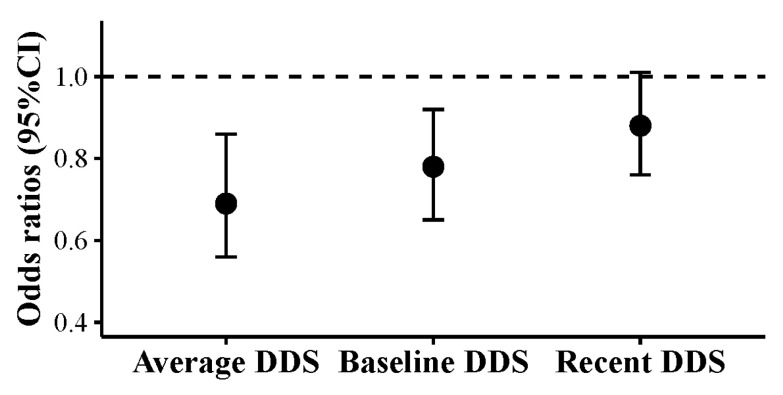
Dietary diversity score (DDS) at different surveys and disability in activities of daily living (ADL). Participants involved in three or more dietary surveys were included (*n* = 2756). Average DDS was the cumulative mean DDS from baseline to the phase prior to the end of the survey (report of disability in activities of daily living, loss to follow-up, the phase of 2015, whichever occurred first). Baseline DDS was obtained from the phase at entry. Recent DDS was obtained from the phase before the end of the survey. All DDSs were continuous. Odds ratios were estimated by logistic regression models. Models were adjusted for age at entry (continuous), gender (men or women), living region (southern or northern China), residency (urban or rural), income (low, middle, or high), education level (primary school and below or middle school and higher), smoking status (smoker or not), physical activity (≤100 or >100 metabolic equivalent of task-hours/week), body mass index (continuous), and comorbidities (no or yes).

We found both the average DDS across phases and the baseline DDS were associated with lower odds of ADL disability. The association was more pronounced for the average DDS. The association between the recent DDS prior to the end of the survey and ADL disability was insignificant (Figure 3). 

## 4. Discussion

This study found that higher DDS was inversely associated with the risk of ADL disability in Chinese adults. The association was stable after the adjustment of physical activity, BMI, and comorbidity. To our best knowledge, the present study is the first one revealing the beneficial effect of dietary diversity on ADL disability.

In the western population, the Mediterranean diet is most frequently investigated when addressing the effect of dietary patterns on functional capacity. A meta-analysis showed that higher adherence to the Mediterranean diet is associated with decreased risk of frailty and functional disability in the elderly [25]. Besides, adherence to the healthy eating index may be also associated with better physical performance among elderly people [26]. In the eastern population, prospective studies found the Japanese diet was inversely associated with functional disability in Japanese individuals aged 65 years and above [27,28]. In contrast, evidence on DDS and ADL or functional capacity is relatively limited. We only observed one study that reported an insignificant relationship between DDS and higher-level functional capacity [29]. Our study found an inverse association between DDS and the risk of ADL disability based on a population-based cohort study. We believe this work will make contributions to literature on healthy aging. Besides, compared with the approach of dietary pattern assessment, the DDS approach is easier to compute, more suitable for comparison across different populations, and more appropriate to use for guiding people to follow a healthy diet. This work will contribute to the prevention of ADL disability. 

Our study observed that each additional point on DDS was associated with a nearly thirty percent reduction in the risk of ADL disability. The benefits of DDS on ADL might be related to the following mechanisms. First, aging is associated with a loss of muscle mass, leading to frailty, sarcopenia, and functional disability [30]. More dietary protein is needed for the maintenance of good muscle function in the elderly [30,31]. Epidemiological studies showed that higher dietary protein may slow down the process of muscle mass loss [32,33,34]. Our study found individuals with higher DDS had higher intakes of protein, which may be a benefit for ADL independence. Second, it has been widely recognized that inflammation and oxidative stress played important roles in the process of aging [35,36]. We observed a positive trend between DDS and intakes of antioxidants (e.g., vitamin E, vitamin C, selenium) among the study population. Higher DDS may promote healthier aging by countering inflammation and oxidative stress. Third, aging is also caused by the loss of bone mass [37], which may increase the risk of frailty and fracture and eventually accelerate the loss of ADL independence [38,39,40]. In this study, we observed that participants with higher DDS enjoyed high intakes of protein, calcium, phosphorus, potassium, which were beneficial to bone health [41]. Fourth, a diverse diet has a positive effect on gut microbiota [42]. Healthy gut microbiota may promote the absorption of micronutrients and modulate individual response to dietary protein [31,43]. 

An interaction between DDS and comorbidity on the risk of ADL disability was observed in this study. The association of DDS with ADL disability was more pronounced among participants without comorbidity at baseline than those with comorbidity. We assumed that this might be because, among participants with comorbidity, the progression of disease played a more important role in the loss of ADL independence than the impact of diet. However, we should note that, even in individuals with comorbidity, the beneficial effect of DDS still existed.

In this study, we calculated the cumulative average DDS to address participants’ long-term dietary exposure. To compare the difference between different dietary exposure measures, we estimated the OR for the average DDS across phases, the baseline DDS, and the recent DDS prior to the end of the survey in relation to ADL disability. The results indicate that when estimating the effect of DDS on ADL disability, the average DDS is the most pronounced of the three approaches, which is consistent with previous studies [44,45]. The result indicates that, in estimating the effect of dietary factors on ADL, long-term dietary exposure is more important than the recent exposure. Findings also implicated that the measure that could address the dynamic dietary exposure is more preferable to the single baseline data. 

The strengths of this study include prospective design and population-based samples, which provided advantages for causal inference. Repeated dietary surveys allowed us to capture participants’ long-term dietary exposure. There are several limitations. First, although comorbidities were considered in our analysis, other factors that may influence ADL (e.g., dementia, accident) were not included because of a lack of data. Second, the present study was based on a dynamic cohort study, participants joined the survey at a wide age range; however, in the subgroup and sensitivity analyses, we took participants’ age at entry and phase at entry into consideration, which could partly mark up for this defect. Third, the ADL disability was self-reported. Participants might overestimate or underestimate their abilities to finish some tasks because of social desirability or misunderstanding.

## 5. Conclusions

In summary, this study found that higher DDS has a beneficial effect on the risk of ADL disability and long-term dietary exposure is more preferable in the investigation of DDS and ADL.

## Figures and Tables

**Table 1:** Baseline characteristics of participants according to dietary diversity score (DDS) tertiles.

Variables	DDS Tertiles ^a^			*p*
	T1	T2	T3	
Number of participants	1663	1663	1678	
Age at entry (years)	57.6 ± 9.6	58.1 ± 9.8	60.3 ± 8.9	<0.001
Body mass index (kg/m^2^)	22.2 ± 3.3	23.1 ± 3.4	24.2 ± 3.3	<0.001
Gender				
Men	44.4	48.2	49.0	0.016
Women	55.6	51.8	51.0	
Living region				
Southern China	57.7	71.9	63.5	<0.001
Northern China	42.3	28.1	36.5	
Residency				
Rural	82.9	61.3	30.5	<0.001
Urban	17.1	38.7	69.5	
Education level				<0.001
Primary school or below	86.0	70.8	40.0	
Middle school or higher	14.0	29.2	60.0	
Income ^b^				
Low	51.7	26.8	9.8	<0.001
Middle	31.6	36.7	23.4	
High	16.7	36.5	66.9	
Smoking status				
Smoker	32.7	31.7	25.4	<0.001
Non-smoker	67.3	68.3	74.6	
Alcohol consumption				
Regular drinker	23.4	26.7	24.9	0.101
Others	76.6	73.3	75.1	
Physical activity (MET-hours per week)				
≤100	40.4	55.3	64.7	<0.001
>100	59.6	44.7	35.3	

MET, metabolic equivalent of task. Continuous variables were presented as means and standard deviations, and categorical variables were presented as percentages. Continuous variables were tested between groups by one-way analysis of variance, and categorical variables were tested by chi-square test. ^a^ DDSs were grouped into tertiles from low to high (T1, T2, T3). ^b^ Per capita household incomes were grouped into tertiles at each phase and was labeled as low, middle, and high, respectively.

**Table 2:** Nutrient intakes across dietary diversity score (DDS) tertiles.

Nutrients	DDS Tertiles ^a^		
	T1	T2	T3
Protein (g/d)	55.3 (45.6, 66.2)	61.1 (51.7, 71.8)	69.1 (58.2, 82.8)
Fat (g/d)	55.9 (42.8, 72.2)	73.2 (58.1, 92.3)	81.1 (64.1, 100.0)
Carbohydrate (g/d)	329.3 (265.4, 390.7)	286.4 (233.7, 340.6)	252.2 (202.0, 302.5)
Insoluble dietary fiber (g/d)	10.8 (8.0, 14.2)	9.4 (7.1, 12.5)	11.0 (8.2, 14.7)
Vitamin A (μgRE/d)	263.1 (156.0, 418.3)	391.0 (255.7, 627.4)	473.2 (323.5, 696.1)
Thiamin (mg/d)	0.9 (0.7, 1.1)	0.9 (0.7, 1.0)	0.9 (0.7, 1.1)
Riboflavin (mg/d)	0.6 (0.5, 0.7)	0.7 (0.6, 0.8)	0.8 (0.7, 1.0)
Niacin (mg/d)	12.2 (9.7, 14.8)	13.7 (11.0, 16.5)	14.5 (11.6, 17.8)
Vitamin C (mg/d)	74.6 (52.9, 100.1)	74.9 (55.2, 99.8)	80.2 (57.5, 108.0)
Vitamin E (mg/d)	26.8 (18.0, 37.3)	26.9 (19.5, 37.6)	31.0 (22.6, 41.9)
Calcium (mg/d)	315.8 (245.6, 393.1)	347.1 (275.3, 442.1)	453.8 (350.5, 591.0)
Phosphorus (mg/d)	871.0 (701.7, 1058.0)	875.1 (738.6, 1027.7)	979.7 (832.6, 1158.6)
Potassium (mg/d)	1459.6 (1183.6, 1764.2)	1483.7 (1232.7, 1767.2)	1748.8 (1445.2, 2103.1)
Sodium (mg/d)	5066.2 (3869.7, 6714.2)	4781.3 (3693.8, 6378.6)	4637.7 (3532.3, 6133.1)
Magnesium (mg/d)	283.5 (227.7, 346.2)	266.1 (222.1, 318.9)	286.0 (236.3, 341.0)
Iron (mg/d)	19.5 (15.6, 24.0)	19.5 (16.1, 23.5)	20.5 (16.6, 25.0)
Zinc (mg/d)	9.6 (7.9, 11.5)	10.2 (8.5, 12.1)	10.7 (8.8, 12.8)
Selenium (μg/d)	30.9 (23.0, 39.1)	35.9 (29.4, 45.1)	46.0 (36.7, 57.6)
Copper (mg/d)	1.9 (1.5, 2.3)	1.7 (1.4, 2.1)	1.8 (1.4, 2.3)
Manganese (mg/d)	6.3 (5.0, 7.6)	5.7 (4.6, 6.9)	5.2 (4.2, 6.4)

Values are medians and quartiles. Tests for linear trend across DDS tertiles were conducted by assigning the midpoint values of DDS and treating the variables as continuous in a linear regression model, prior to that, the values of nutrient intakes were transformed to log to reach normality. All nutrients were associated with DDS tertiles with *p*-trend < 0.001, except for dietary fiber (*p*-trend = 0.041), thiamin (*p*-trend = 0.260), magnesium (*p*-trend = 0.033), and copper (*p*-trend = 0.138). ^a^ DDSs were grouped into tertiles from low to high (T1, T2, T3).

**Table 3:** Association between dietary diversity score (DDS) and disability in activities of daily living (ADL).

	Continuous DDS	DDS Tertiles ^a^			
		T1	T2	T3	*p*-Trend
*n* of ADL disability	601	281	194	126	
*n* of person years	52,297	19,458	18,414	14,425	
Crude	0.86 (0.78, 0.94)	1.00	0.82 (0.68, 0.98)	0.74 (0.60, 0.91)	0.003
Model 1	0.73 (0.65, 0.82)	1.00	0.81 (0.67, 0.99)	0.53 (0.41, 0.69)	<0.001
Model 2	0.71 (0.63, 0.80)	1.00	0.80 (0.65, 0.97)	0.50 (0.39, 0.66)	<0.001

Values were hazard ratios and 95% confidence intervals unless specified. Hazard ratios were estimated by Cox proportional regression models. Multivariate models were adjusted for: Model 1: age at entry (continuous), gender (men or women), living region (southern or northern China), residency (urban or rural), income (low, middle, or high), and education level (primary school and below or middle school and higher); Model 2: additionally included smoking status (smoker or not), physical activity (≤100 or >100 metabolic equivalent of task-hours/week), body mass index (continuous), and comorbidities (no or yes). Tests for trend were performed by assigning the midpoints of each DDS tertiles and treating the value as continuous in a separate regression model. ^a^ DDSs were grouped into tertiles from low to high (T1, T2, T3).

## Supplementary Materials

The following are available online at https://www.mdpi.com/2072-6643/12/11/3263/s1, Table S1: Sensitivity analysis of association of dietary diversity score (DDS) with disability in activities of daily living (ADL).
