# Supplementary material for: Beneficial Effect of Dietary Diversity on the Risk of Disability in Activities of Daily Living in Adults: A Prospective Cohort Study
Source: Nutrients. 2020 Oct 25;12(11):3263. doi: 10.3390/nu12113263 (PMC7692387; doi:10.3390/nu12113263)
Supplement: Supplementary file 1 [file nutrients-12-03263-s001.pdf]

Table S1. Sensitivity analysis of association of dietary diversity score (DDS) with disability in activities of daily living (ADL).

	**Continuous DDS**	**DDS tertiles [a]**			
		**T1**	**T2**	**T3**	***p*-trend**
Model 1	0.78(0.67,0.91)	1.00	0.83(0.65,1.06)	0.55(0.39,0.76)	<0.001
Model 2	0.73(0.64,0.82)	1.00	0.82(0.67,1.01)	0.52(0.39,0.68)	<0.001

Values are hazard ratios and 95% confidence intervals unless specified. Hazard ratios were estimated by Cox proportional regression models. Models were adjusted for age at entry (continuous), gender (men or women), living region (southern or northern China), residency (urban or rural), income (low, middle, or high), education level (primary school and below or middle school and higher), smoking status (smoker or not), physical activity (≤100 or >100 metabolic equivalent of task-hours/week), body mass index (continuous), and comorbidities (no or yes). Tests for trend were performed by assigning the midpoints of each dietary diversity score tertiles and treating the value as continuous in a separate regression model. Model 1 was conducted by excluding participants whose follow-up time was less than 5 years. Model 2 was conducted by additional adjustment of alcohol consumption (regular consumer or not) and wave at entry (1997, 2000, 2004, 2006, 2009, or 2011). [a] DDSs were grouped into tertiles from low to high (T1, T2, T3).
